# Valid Probabilistic Predictions for Ginseng with Venn Machines Using Electronic Nose

**DOI:** 10.3390/s16071088

**Published:** 2016-07-13

**Authors:** You Wang, Jiacheng Miao, Xiaofeng Lyu, Linfeng Liu, Zhiyuan Luo, Guang Li

**Affiliations:** 1 State Key Laboratory of Industrial Control Technology, Institute of Cyber Systems and Control, Zhejiang University, Hangzhou 310027, Zhejiang, China; king_wy@zju.edu.cn (Y.W.); jiacheng@zju.edu.cn (J.M.); xxlm@zju.edu.cn (X.L.); liulinfengzju@zju.edu.cn (L.L.); 2 Computer Learning Research Centre, Royal Holloway, University of London, Egham Hill, Egham, Surrey TW20 0EX, UK; zhiyuan@cs.rhul.ac.uk

**Keywords:** electronic nose, ginseng, Venn machine, probabilistic prediction, support vector machine

## Abstract

In the application of electronic noses (E-noses), probabilistic prediction is a good way to estimate how confident we are about our prediction. In this work, a homemade E-nose system embedded with 16 metal-oxide semi-conductive gas sensors was used to discriminate nine kinds of ginsengs of different species or production places. A flexible machine learning framework, Venn machine (VM) was introduced to make probabilistic predictions for each prediction. Three Venn predictors were developed based on three classical probabilistic prediction methods (Platt’s method, Softmax regression and Naive Bayes). Three Venn predictors and three classical probabilistic prediction methods were compared in aspect of classification rate and especially the validity of estimated probability. A best classification rate of 88.57% was achieved with Platt’s method in offline mode, and the classification rate of VM-SVM (Venn machine based on Support Vector Machine) was 86.35%, just 2.22% lower. The validity of Venn predictors performed better than that of corresponding classical probabilistic prediction methods. The validity of VM-SVM was superior to the other methods. The results demonstrated that Venn machine is a flexible tool to make precise and valid probabilistic prediction in the application of E-nose, and VM-SVM achieved the best performance for the probabilistic prediction of ginseng samples.

## 1. Introduction

Electronic noses (E-noses) are devices designed to precisely detect and distinguish volatiles in complex samples by mimicking the mammalian sensory system. After thirty years of development, E-noses has been applied in the field of environmental quality monitoring [1], food and beverage quality control [2,3,4], medical diagnosis [5,6,7] and others.

Ginseng (*Panax ginseng* C.A. Meyer, mainly cultivated in Korea and Northeast China) and American ginseng (*Panax quinquefolius*, mainly cultivated in North America and China) are two main categories of ginseng, which can be subdivided into different subcategories according to species and production places Ginsengs are widely used in Chinese traditional medicine with great medicinal value. The increasing demand and consumption of ginsengs have led to some species substitution or adulteration with other species because of the high profits involved. However, it is not easy to identify the origin of ginseng species just based just on their morphology. Besides, many products are provided commercially in the form of a powder or shredded slices, which increases the difficulties of identification. Traditional identification of ginsengs is implemented with sensory analysis by a panel of experts, which is a costly process. Meanwhile, the validation of identification only depends on the various levels of experts’ experience. Normal analytical techniques, such as gas chromatography-mass spectrometry (GC-MS) [8] and high performance liquid chromatography (HPLC) [9] require long analysis times, complicated sample pretreatment and the use of sophisticated equipment. In this sense, electronic noses are a promising analysis system for the identification of traditional Chinese medicines due to their precise and stable performance, simple sample pretreatment, short measurement time and low cost.

Classification is one of major types of data treatment used for E-noses, which is also one of standard subjects in machine learning. In the applications of medical diagnosis, military usage and others, only predicting the label of testing sample is not enough, the probability estimation, which measure our trust in the prediction, should also be provided since it provides important information for risk control. Probabilistic prediction methods can help solve the problem.

Several classical probabilistic prediction algorithms have been proposed and widely used for classification cases, including Platt’s method [10] (based on support vector machine, SVM), logistic regression (for binary case) or Softmax regression (SR, for multi-classification case), Naive Bayes classification (NB) [11] and etc. However, most of these methods are based on strong statistical assumptions about the example distribution. Usually, examples distribution in Naïve Bayes was assumed to be a Gaussian distribution. Softmax Regression assumes the estimated probability to be the specific mathematic form shown in Section 2.3. Platt’s method assumes pairwise class probabilities take the specific mathematic form that will be shown in Section 2.4. Hence, once the assumed statistical model is unavailable or even incorrect, the estimated probability will be biased and the classification rate will be poor. This case can easily happen to E-nose data, because usually it’s difficult to find an accurate statistical model for real-world data.

The validity of probabilistic prediction is very important for probabilistic prediction methods. Validity means the estimated probability for predicted label is unbiased, which means the estimated probability is equal or close to the observed frequency that predictions are correct.

In this work, a flexible machine learning framework, Venn machine [12,13,14] was introduced to make valid probabilistic prediction for different species of ginseng samples, which means the estimated probability is unbiased. The only assumption required for Venn machine is that the example distribution is an independent identical distribution (I.I.D assumption), which can be easily satisfied by E-nose data, and do not need specific distribution of E-nose data Once the I.I.D assumption is satisfied, the validity of predicted probabilities is guaranteed.

A homemade electronic nose embedded with a sensor array of 16 metal-oxide semi-conductive gas sensors was used for the discrimination of nine ginsengs of different species or production places. Three Venn predictors separately based on three classical probabilistic prediction methods (Platt’s method, Softmax regression and Naive Bayes classification) and these three classical probabilistic prediction methods were developed to make probabilistic predictions for the ginseng samples. The performance of six methods were compared in the aspect of classification rate and validity of probabilistic predictions in offline mode. The validity and precision of probabilistic prediction by Venn predictors in online mode were also investigated. Electronic nose with VM-SVM demonstrated its strong ability to make precise and valid probability estimate with good classification rate.

## 2. Probabilistic Prediction Methods

### 2.1. Venn Machine

Consider a training set $z_{i}=\left(x_{i},y_{i}\right),i=1,\ldots ,n-1$, consists of objects $x_{i}\in X$ and its label $y_{i}\in Y$, The task is to predict $y_{n}$ for a new object $x_{n}$ and estimate the probability that the prediction is correct.

Firstly, hypothesizing $y_{n}=y,y\in Y$. y is finite, and for every attempt $\left(x_{n},y\right)$, a Venn taxonomy, which is a measurable function $A_{n}$, n∈N, divide each example in $\left\{z_{1},\ldots ,z_{n}\right\}$ into the finite categories $t_{i}$, $t_{i}\in T$ (T is the set of all sample categories) as follows:

(1) $$t_{i}=A_{n}\left(\left\{z_{1},\ldots ,z_{i-1},z_{i+1},\ldots ,z_{n}\right\},z_{i}\right),i=1,\ldots ,n$$

$z_{i}$ and $z_{j}$ are assigned to the same category if and only if:

(2) $$A_{n}\left(\left\{z_{1},\ldots ,z_{i-1},z_{i+1},\ldots ,z_{n}\right\},z_{i}\right)=A_{n}(\left\{z_{1},\ldots ,z_{j-1},z_{j+1},\ldots ,z_{n}\right\},z_{j})$$

Let $p_{y}$ be the empirical probability distribution of the labels in category τ, where τ is the category that $z_{n}$ is divided to:

(3) $$p_{y}\left\{y^{\prime }\right\}=\frac{\left|\left\{(x^{*},y^{*})\in \tau :y^{*}=y^{\prime }\right\}\right|}{\left|\tau \right|}$$

|τ| is the number of examples in category τ. $p_{y}$, which is a row vector, is a probability distribution on Y consisting of *K* probabilities, where K=|Y|. After trying all *y*, we get a K×K matrix *P*. We define the minimum entry of a column as the quality of the column. The best column with the highest quality $j_{\mathrm{best}}$ is our prediction for $x_{n}$ and the probability interval that the prediction is correct is:

(4) $$\left[\mathrm{Pl},\mathrm{Pu}\right]=[\underset{i=1,\ldots ,K}{\min}P_{i,j_{\mathrm{best}}},\underset{i=1,\ldots ,K}{\max}P_{i,j_{\mathrm{best}}}]$$

where Pl and Pu is the lower and upper bound of the probability interval.

Any classical classification algorithm can be used as Venn taxonomy $A_{n}$ to assign a category *t* to $z_{i}$ with a training set $\left\{z_{1},\ldots ,z_{i-1},z_{i+1},\ldots ,z_{n}\right\}$. The validity of Venn machine is guaranteed in theory if the assumption that set $z_{i}=\left(x_{i},y_{i}\right),i=1,\ldots ,n$ is generated from independent identical distribution is satisfied.

To further clarify how a Venn machine works, a simple example is given. Hypothesizing a dataset consisting of three categories labelled with A, B and C with 30 samples, 10 samples in each category. The aim is to make probabilistic prediction for new sample x with unknown label y.

Firstly, setting y = A, new sample (x, A) was added to the dataset, and total number of samples was 31. Then leave-one-out cross-validation was applied to predict the label for each sample. Finally, 31 prediction were obtained. Hypothesizing the label of x is predicted to be B, also 1 sample with true label A, 7 samples with true label B and 1 sample with true label C were also predicted to be B. then we got a row vector $p_{A}=[1/9\;7/9\;2/9]=[0.11\;0.78\;0.11]$.

Secondly, setting y = B, then new sample (x, B) were added to the dataset. Leave-one-out cross-validation was applied to predict the label of each sample. Hypothesizing we got $p_{B}=[0.00\;0.91\;0.09]$. Thirdly, setting y = C, then new sample (x, C) were added to the dataset. Leave-one-out cross-validation was applied to predict the label of each sample. Hypothesizing we got $p_{c}=[0.10\;0.70\;0.20]$.

Finally, we got matrix P:

$$P=\left[\begin{array}{ccc} 0.11 & 0.78 & 0.11\\ 0.00 & 0.91 & 0.09\\ 0.10 & 0.70 & 0.20 \end{array}\right]$$

$j_{\mathrm{best}}$ is B with best quality of the second column being 0.70. The predicted label for new sample x is B with probability interval [0.70 0.91].

### 2.2. Naive Bayes Classification

The Naïve Bayes classifier is designed to be used when features are independent of one another within each class. It appears to still work well in some cases when the independence assumption is not satisfied. It is based on Bayes’ theorem and defined the probability that example *x* belong to class k, k = 1,..., K is:

(5) $$P\left(y=k|x\right)=\frac{P\left(y=k\right)P\left(x|y=k\right)}{P\left(x\right)}=\frac{P(y=k)\prod_{j=1}^{m}P(x_{j}|y=k)}{P\left(x\right)}$$

Appropriate specific distribution should be assumed for the probability density $P(x_{j}|y=k)$ of examples and the Gaussian distribution is most widely used.

### 2.3. Softmax Regression

Softmax regression is a generalization of logistic regression for multi-classification. Given a test input *x*, to estimate the probability that P(y=k|x) for each class k = 1, …, K, we assume the K estimated probability take the form:

(6) $$h_{\theta }\left(x\right)=\left[\begin{array}{c} P\left(y=1|x;\theta \right)\\ P\left(y=2|x;\theta \right)\\ \vdots \\ P\left(y=k|x;\theta \right) \end{array}\right]=\frac{1}{\sum_{j=1}^{k}\exp\left(\theta {\left(j\right)}^{T}x\right)}\left[\begin{array}{c} \exp\left(\theta {\left(1\right)}^{T}x\right)\\ \exp\left(\theta {\left(2\right)}^{T}x\right)\\ \vdots \\ \exp\left(\theta {\left(k\right)}^{T}x\right) \end{array}\right]$$

where θ(1), θ(2), …, θ(k) are parameter in the model needed to be optimized. The optimization objective is to minimize J(θ):

(7) $$\min J\left(\theta \right)=-\left[\overset{l}{\underset{i=1}{\sum }}\overset{K}{\underset{t=1}{\sum }}1\left\{y\left(i\right)=t\right\}\log\frac{\exp\left(\theta {\left(t\right)}^{T}x\left(i\right)\right)}{\sum_{j=1}^{k}\exp\left(\theta {\left(j\right)}^{T}x\left(i\right)\right)}\right]$$

### 2.4. Platt’s Method

Platt’s method was proposed to make SVM output probabilistic prediction [15]. An improved implementation was finished by Lin et al. [10]. Given a test input *x*, to estimate the probability that P(y=k|x) for each class k, k = 1, …, K, we firstly estimate pairwise class probabilities:

(8) $$r_{\mathrm{ij}}\approx P\left(y=i|y=i\;\mathrm{or}\;j,x\right)$$

If *f* is the decision value at x, then assuming pairwise class probabilities take the form:

(9) $$r_{\mathrm{ij}}\approx \frac{1}{1+e^{\mathrm{Af}+B}}$$

where A and B are estimated by:

$$\underset{A,B}{\min}\underset{y_{l}=i\;\mathrm{or}\;j}{\sum }1\left\{y_{l}=i\right\}\cdot r_{\mathrm{ij}}+1\left\{y_{l}=j\right\}\cdot r_{\mathrm{ji}}$$

where 1{a true statement}=1, 1{a false statement}=0*.* After collecting all $r_{\mathrm{ij}}$ values, the following optimization problem is solved as:

$$\underset{p}{\min}\frac{1}{2}\overset{k}{\underset{i=1}{\sum }}\underset{i:j\neq i}{\sum }{\left(r_{\mathrm{ji}}p_{i}-r_{\mathrm{ij}}p_{j}\right)}^{2}$$

$$\mathrm{Subject}\;\mathrm{to}\;p_{i}\geq 0,i=1,\ldots ,k,\;\overset{k}{\underset{i=1}{\sum }}p_{i}=1$$

### 2.5. The Validity of Probabilistic Predictions

Probabilistic prediction can provide reliability estimate on the prediction. However, the estimated probability should be valid. In this work, we used two methods to examine the validity of probabilistic predictions. One is to use the standard loss function, the other one is to compare the cumulative probability value for predictions with cumulative number of correct predictions.

#### 2.5.1. Loss Function

Two loss functions, log loss and square loss, were applied in this paper. Supposing *p* is the probability value for predicted label of testing example *x*. *q* is equal to 1 if the prediction is correct and equal to 0 otherwise. The log loss function is:

(10) $$\lambda_{\ln}\left(p,q\right)=\{\begin{array}{c} -\ln\left(1-p\right)\;\mathrm{if}\;q=0\\ -\ln p\;\mathrm{if}\;q=1 \end{array}$$

The square loss function is:

(11) $$\lambda_{\mathrm{sq}}\left(p,q\right)={\left(p-q\right)}^{2}$$

However, Venn machine outputs a probability interval (Pl, Pu) instead of single probability value *p*. So the corresponding minimax probabilistic prediction *p* is defined as follows: 

For log loss function:

(12) $$p=\frac{p_{u}}{1-p_{l}+p_{u}}$$

For square loss function:

(13) $$p=p_{u}+\frac{{p_{l}}^{2}}{2}-\frac{{p_{u}}^{2}}{2}$$

$p\in (p_{l},p_{u})$. For more details about the derivation of Equations (12) and (13), please refer to the work [16]. Given n testing examples, for different methods, we will calculate and compare the mean log error ($d_{\ln}$):

(14) $$d_{\ln}=\frac{1}{n}\overset{n}{\underset{i=1}{\sum }}\lambda_{\ln}\left(p_{i},q_{i}\right)$$

and the root mean square error ($d_{\mathrm{sq}}$):

(15) $$d_{\mathrm{sq}}=\frac{1}{n}\overset{n}{\underset{i=1}{\sum }}\lambda_{\mathrm{sq}}\left(p_{i},q_{i}\right)$$

For each method, the smaller the $d_{\ln}$ and $d_{\mathrm{sq}}$ are, the better the validity of the method is.

#### 2.5.2. Cumulative Probability Values versus Cumulative Correct Predictions

If the probability estimated by certain method is valid, with large number of *n* testing examples, the cumulative probability value (CP) should be close or equal to cumulative number of correct predictions (CN):

(16) $$\overset{n}{\underset{i=1}{\sum }}p(y_{i}^{*})\approx \overset{n}{\underset{i=1}{\sum }}1_{i}\left\{y_{i}^{*}=y_{i}\right\}$$

$y_{i}^{*}$ is the predicted label for $x_{i}$, and $y_{i}$ is the true label. So the second assessment criteria for the validity of probabilistic prediction is defined to be the absolute difference between the average probability value and the ratio of correct prediction:

(17) $$d_{1}=\left|\frac{\sum_{i=1}^{N}1_{i}\left\{y_{i}^{*}=y_{i}\right\}}{N}-\frac{\sum_{i=1}^{N}p\left(y_{i}^{*}\right)}{N}\right|$$

However, for Venn machines, which outputs a probability interval (Pl, Pu) instead of a single probability value, the cumulative number of correct predictions should be between the cumulative lower probability and the cumulative upper probability. This is an overall estimate on the validation of probabilistic prediction methods:

(18) $$\overset{n}{\underset{i=1}{\sum }}\mathrm{Pl}(y_{i}^{*})\leq \overset{n}{\underset{i=1}{\sum }}1_{i}\left\{y_{i}^{*}=y_{i}\right\}\leq \overset{n}{\underset{i=1}{\sum }}\mathrm{Pu}(y_{i}^{*})$$

Firstly, we calculated the absolute difference between ratios of correct predictions, with cumulative upper bounds of probability intervals:

(19) $$d_{11}=\left|\frac{\sum_{i=1}^{N}1_{i}\left\{y_{i}^{*}=y_{i}\right\}}{N}-\frac{\sum_{i=1}^{N}\mathrm{pl}\left(y_{i}^{*}\right)}{N}\right|$$

and with cumulative lower bounds of probability intervals:

(20) $$d_{12}=\left|\frac{\sum_{i=1}^{N}1_{i}\left\{y_{i}^{*}=y_{i}\right\}}{N}-\frac{\sum_{i=1}^{N}\mathrm{pu}\left(y_{i}^{*}\right)}{N}\right|$$

Then, the second assessment criteria for the validity of probabilistic predictions by Venn machine is defined as follows:

(21) $$d_{1}=\max\left(d_{11},d_{12}\right)$$

For each method, the smaller $d_{1}$ is, the better the validity of the method is.

## 3. Materials and Methods

### 3.1. Sample Preparation

Nine categories of ginsengs, thirty-five pieces of ginseng roots for each category, were randomly purchased from Changchun Medicinal Material Market (Changchun, China) and the details of materials were shown in Table 1. Prior to E-nose measurement, every ginseng root was ground into powder separately, and 10 g powder of each sample was placed and sealed in 100 mL headspace vials. Then the vials were placed in a thermostat at 50 °C for 30 min. 10 ml headspace gas from each vial was extracted with s syringe for measurement.

### 3.2. E-Nose Equipment and Measurement

A homemade E-nose embedded with sensor array of 16 metal-oxide gas sensors, was used in our experiment. The schematic diagram of the E-nose system was introduced in early work [17]. The gas sensors were purchased from Figaro Engineering Inc. (Osaka, Japan), including TGS800, TGS813*2, TGS816, TGS821, TGS822*2, TGS826, TGS830, TGS832, TGS880, TGS2600, TGS2602, TGS2610, TGS2611, TGS2620. All sensors were fixed on a printed circuit board, which was placed in a 200 mL stainless chamber. A three-way valve (Chengdu Qihai Electromechanical Equipment Manufacturing Co. Ltd., Chengdu, China) was designed to switch between target gas and clean dry air. Two mini vacuum pumps (Chengdu Qihai Electromechanical Equipment Manufacturing Co. Ltd.) were used for gas washing at a constant flow of 1 L/min. A data acquisition (DAQ) unit USB6211, purchased from National Instrument Inc. (Austin, TX, USA), was equipped to acquire the signal of sensors and control pumps. Heater voltage of 5V DC, recommended by Figaro Inc., was applied for each sensor to attain the best performance of sensor.

The measurement process was as follows: the chamber loaded with the sensor array was washed with a clean-dry-air flow of 1 L/min for 360 s to allow the sensors to return to baseline. Then the air flow was stopped and the target gas was injected into the chamber with a syringe. The responses of the sensors lasted for 180 s and then the air flow was turned on to wash away the target gas. The signals of 16 sensors were recorded for 340 s at 2 Hz for one measurement, including 20 s before and 140 s after the responses of sensors. The experiment was conducted at room temperature of 20–25 °C and humidity of 50%–70%. Finally, 9 × 35 = 315 samples were obtained (nine categories, 35 samples for each category).

### 3.3. Data Processing

**Figure 1:**
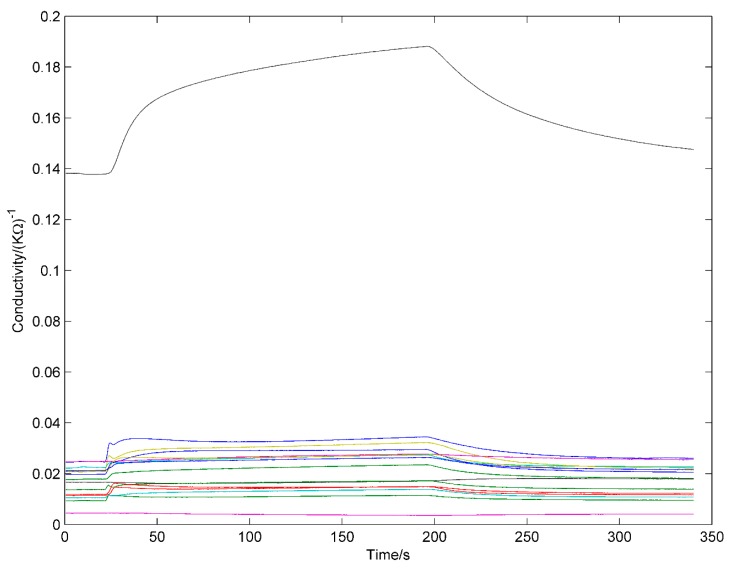
Typical responses of 16 sensors to ginseng samples.

A typical response curves of 16 sensors to ginseng samples were shown in Figure 1. Firstly, the voltage signal of each sensor was calibrated separately by:

(22) $$V=V_{s}-V_{o}$$

where $V_{s}$ is resistance signal, $V_{o}$ is the baseline.

Then, eight common used features were extracted from each sensor as follows:

1. The maximal absolute response, $V_{\max}=\max\left(\left|V\right|\right)$, which is most efficient and widely-used steady feature.
2. The area under the full response curve, $V_{\mathrm{int}}=\int_{0}^{T}V(t)\mathrm{dt}$, where T (=340 s) is the total measurement time, which is also widely- used steady feature.
3–8. Exponential moving average of derivative [18,19] of V, $E_{a}\left(V\right)=[\min\left(y\left(k\right)\right),\max\left(y\left(k\right)\right)$, where the discretely sampled exponential moving average y(k)=(1−a)y(k−1)+a(V(k)−V(k−1)) with smoothing factors a=1/(100×SR), 1/(10×SR), 1/SR. SR is the sampling rate, SR = 10 Hz. y(1)=aV(1). For each smoothing factor, two features were extracted. A total of six transient feature were extracted. Besides steady features, transient features were considered to contain much effective information that should be made the best of [20].
  Finally, 16 × 8 = 128 features are extracted from each sample and all the features are scaled to [0 1]:
  (23) $$x_{i,j}^{\prime }=\frac{x_{i,j}-\underset{j}{\min}(x_{i,j})}{\underset{j}{\max(}x_{i,j})-\underset{j}{\min(}x_{i,j})}$$
  where $x_{i,j}$ is the *j*th feature from *i*th sensor. $x_{i,j}^{\prime }$ is the feature after normalization.

Three Venn predictors, VM-SVM, VM-SR and VM-NB, separately based on SVM, Softmax regression and Naïve Bayes, were developed for the probabilistic prediction of ginseng samples in offline and online modes. Three classical probabilistic prediction methods: Platt’s method, Softmax regression and Naïve Bayes were also used for the probabilistic prediction of ginseng samples in offline and online modes. The classification rate and validity of probabilistic predictions by Venn predictors and classical probabilistic prediction methods were compared in offline mode. Platt’s method and SVM were performed with libsvm toolbox [21] using C-SVC (SVM classification with cost parameter C) with nonlinear kernel of Radial Basis Function (RBF). In the training stage, C was searched within [2^2^, 2^4^ … 2^14^] and γ (parameter of kernel RBF) was searched within [2^−9^, 2^−7^ … 2^−1^] with 5-fold cross-validation to obtain optimum parameters for the model. Then, the trained model was performed on the testing examples. For Naïve Bayes, the distribution was set to be Gaussian distribution. All algorithm were implemented in MATLAB 2014a.

## 4. Results and Discussion

In this work, the performance of probabilistic predictors was investigated in both offline and online mode. In offline mode, the dataset was divided in to two parts: training set and testing set. The model was trained with the training set to form a fixed decision rule. Then the model was applied for the testing set. The prediction performance of testing set was treated as criterion of the model. This is the usual way in most works. In online mode, every time one sample was tested, the sample with its true label was added in the training set, then the model was rebuilt and used to predict for another testing sample. As the number of samples in training set increased, the performance of the model improved gradually and tended to be stable. Online prediction is also very meaningful in practical applications. Firstly, we compared the classification rate and validity of six probabilistic predictors in offline mode. Then, we investigated the validity of Venn predictors in online mode.

### 4.1. Performance of Probabilistic Predictors in Offline Mode

In offline mode, three Venn predictors, VM-SVM, VM-SR, VM-NB, and corresponding underlying probabilistic prediction methods, Platt’s method, Softmax Regression, Naïve Bayes were applied for predictions of ginseng samples with ‘leave-one-out’ cross-validation: in every cycle, one sample from sample set was treated as testing set, the other samples were treated as training set. Model of prediction method trained with training set was applied to predict for the testing set. The cycle was repeated until all samples in sample set were treated as testing set once. Then the classification rates and assessment criteria of validity of probabilistic prediction results by each method were investigated and shown in Table 2.

From Table 2, we can see that Platt’s method based on SVM achieved the highest classification rate, and classification rate of VM-SVM was just 2.22% lower than that of Platt’s method. The classification rates of VM-SR were a little higher than that of corresponding underlying algorithm SR. However, Venn machine greatly improved the classification rate of Naïve Bayes from 40% to 60%, which demonstrated the ability of Venn machine to improve the classification performance of the underlying method, though it didn’t work for every method. The assessment criteria of validity of Venn predictors were all smaller than that of corresponding underlying methods, which indicated that the probabilistic prediction conducted by Venn predictors were more valid than corresponding underlying probabilistic prediction methods.

VM-SVM, which benefits from the strong classification performance of SVM, achieved the best classification rate and assessment criteria of validity among three Venn predictors, though $d_{1}$ of VM-SVM was a litter bigger than that of VM-NB. We can conclude that the VM-SVM achieved the best performance among three Venn predictors, VM-SR came next, and VM-NB performed worst because of poor classification ability of Naïve Bayes for ginseng samples. The results indicated that choosing appropriate underlying classification method is very important for Venn predictors.

Similar to binary case, sensitivity and specificity for classification result of dataset with nine category are defined as: for certain category, sensitivity is the proportion that samples in this category are correctly classified, specificity is one minus proportion that samples in other categories are wrongly classified to this category. The sensitivity and specificity for each category with each method were shown in Table 3.

**Figure 2:**
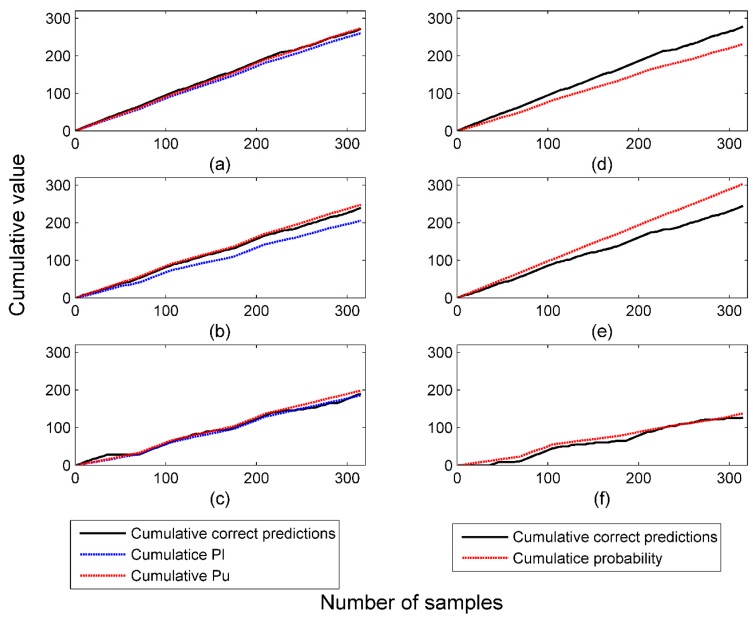
Validity of probabilistic predictions by (**a**) VM-SVM; (**b**) VM-SR; (**c**) VM-NB; (**d**) Platt’s method; (**e**) Softmax Regression; and (**f**) Naïve Bayes in offline mode.

To further investigate the validity of each method, the cumulative probability value/interval was compared with cumulative correct predictions, as shown in Figure 2. We can see that for all three Venn predictors, as the number of predicted samples increase, the cumulative correct predictions kept increasing between cumulative upper bound and cumulative lower bound of probability intervals, with some exceptions caused by statistical fluctuations. This result indicates that the validity of Venn predictors is guaranteed in spite of underlying methods. However, for Platt’s method and Softmax Regression, as the cumulative numbers of predictions increase, the cumulative probability values deviated gradually from cumulative correct predictions. On the whole, the predicted probability values were underestimated by Platt’s method and overestimated by Softmax Regression.

The average widths of probability interval of VM-SVM and VM-NB were 0.0417 and 0.0396 separately, which demonstrated that the probability intervals by Venn predictors were quite narrow and nearly as precise as single probability value by classical probability prediction methods. The average width of probability interval of VM-SR was 0.1353, which was not as good as VM-SVM and VM-NB. This result indicates that the precision of Venn predictors are greatly dependent on underlying classification methods.

All the results above demonstrated the superior performance of the Venn machine over classical probabilistic prediction methods in the aspect of validity of predictions, and VM-SVM is the most reliable probabilistic prediction method for ginseng samples. Platt’s method achieved a little higher classification rate, but worse validity performance than VM-SVM, so we need to take a balance between them in practical application according to different aims.

### 4.2. Performance of Venn Predictors in Online Mode

**Figure 3:**
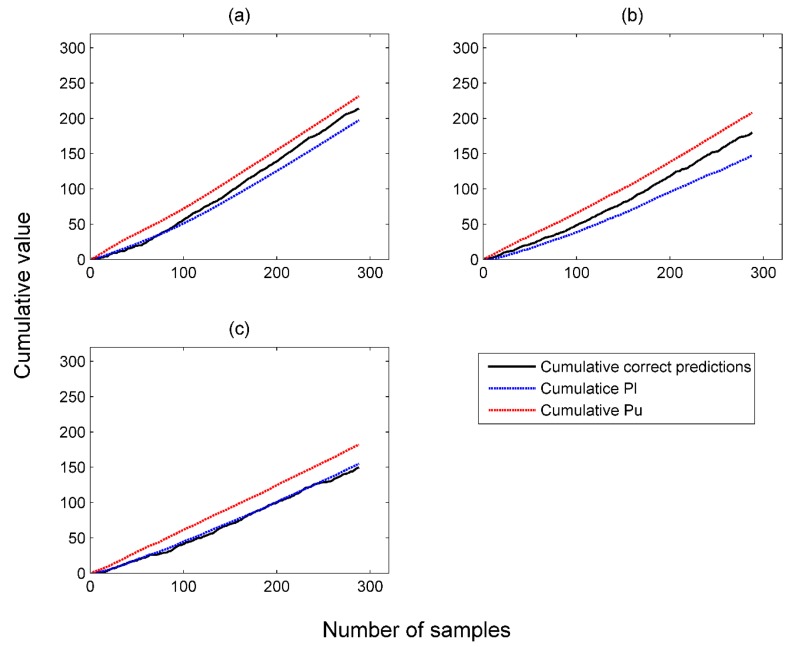
Validity of probabilistic predictions by (**a**) VM-SVM (Venn machine based on SVM); (**b**) VM-SR (Venn machine based on Softmax Regression); (**c**) VM-NB (Venn machine based on Naïve Bayes) in online mode.

In the initial stage of online prediction, the model performance is poor due to the limited number of training samples. As the number of training samples increases, the model performance improves and gradually becomes stable. Whether the validity of the probabilistic prediction still holds and how the precision of the probabilistic prediction changes in online mode were investigated here. In online mode, three samples of each category were randomly taken from the sample set to constitute the initial training set, and the other samples were treated as the testing set. In every cycle, one sample was taken without replacement from the testing set, and the prediction method model was trained with the training set was applied to predict the sample. Then the sample with correct label was added into the training set, then the model was updated and the next sample predicted. The cycle was repeated until all the samples in the testing set were tested. The correct predictions of VM-SVM, VM-SR and VM-NB were accumulated according to the sequence that the samples were tested and compared with the corresponding cumulative upper and lower bound of the probability intervals, as shown in Figure 3. The validity of probability predictions of Venn predictors still held all the way and in spite of the underlying classification method in online mode. Similar to the performance of Venn predictors in offline mode, VM-SVM performed best in online mode, VM-SR came next, VM-NB performed worst.

**Figure 4:**
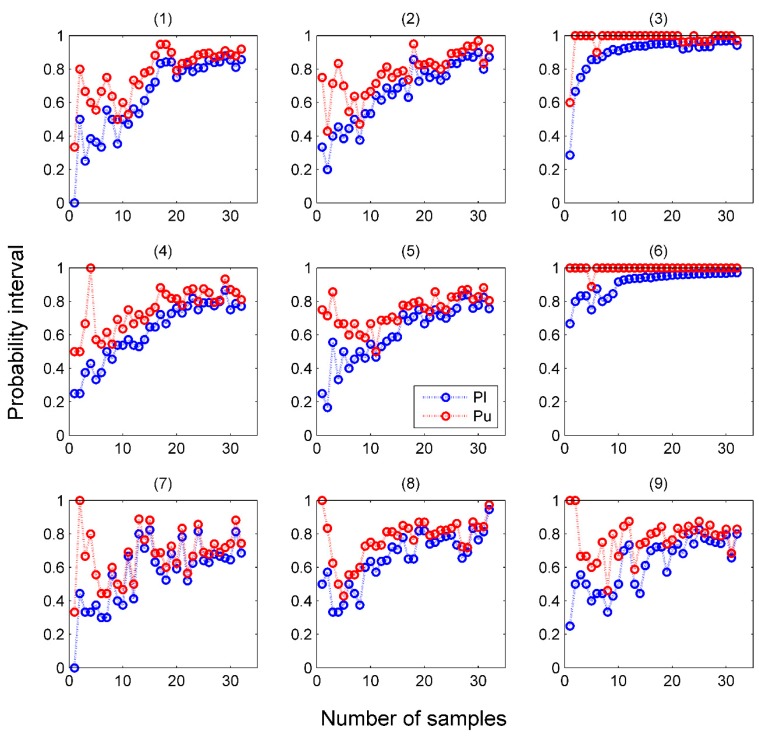
Change of precision of predicted probability intervals for samples from category (**1**–**9**) during online prediction process with VM-SVM.

The validity of Venn predictors was guaranteed, then we investigated the precision of the probability interval and observed the change of probability interval during online prediction process. As the prediction ability of each method for samples from different categories was different, we separately showed the probability intervals of samples from each category according the sequence in which they were tested. Taking the result of VM-SVM as an example, as shown in Figure 4, as the number of the samples in training set increased, the width of probability intervals gradually decreased and the distribution gradually moved upwards, which means the probabilistic predictions yielded by Venn predictors become more and more precise as the number of samples in training set increased while the validity still held all the way.

## 5. Conclusions

In this work, a homemade metal-oxide-sensor-based electronic nose was utilized to discriminate a large set of ginseng samples. A flexible machine learning framework, Venn machine, was introduced to make precise and valid probabilistic prediction for ginsengs. Three Venn predictors were developed and compared with three classical probabilistic prediction methods in the aspect of classification rate and especially the validity of probabilistic predictions. The results showed that the validity of Venn predictors held all the way as the samples increased in spite of the underlying classification methods in both offline and online mode. The validity of Platt’s method and Softmax Regression was much worse than that of corresponding Venn predictors. Platt’s method achieved a best classification rate of 88.57% in offline mod, which was 2.22% higher than that of VM-SVM. The classification rates of VM-SR were a little higher than that of corresponding underlying method. We also surprisingly found that VM-NB greatly improved the classification rate of Naïve Bayes from 40% to 60%. The probability intervals output by Venn predictor were quite narrow, and close to single probability value. The results indicated that Venn machine was a flexible tool for the application of electronic noses which output precise and valid probabilistic predictions.

## Figures and Tables

**Table 1:** Details of the ginseng samples.

No.	Ginseng Species	Places of Production
1	Chinese red ginseng	Ji’an
2	Chinese red ginseng	Fusong
3	Korean red ginseng	Ji’an
4	Chinese white ginseng	Ji’an
5	Chinese white ginseng	Fusong
6	American ginseng	Fusong
7	American ginseng	USA
8	American ginseng	Canada
9	American ginseng	Tonghua

**Table 2:** Classification rates and assessment criteria of validity of probabilistic prediction results for ginseng samples by each method.

Methods	Classification Rate	Assessment Criteria of Validity		
		d_ln_	d_sq_	d_1_
VM-SVM	86.35%	**0.3862**	**0.3419**	**0.0373**
Platt’s method	**88.57%**	0.3876	0.3439	0.1480
VM-SR	**77.78%**	**0.4690**	**0.3938**	**0.1085**
SR	76.19%	Inf ^a^	0.4376	0.1853
VM-NB	**60.32%**	**0.5683**	**0.4475**	**0.0266**
NB	40.32%	0.5851	0.4510	0.0332

^a^ In the prediction result of SR, predicted probability value of certain sample was 1, whereas the prediction was wrong, which lead this criteria to be infinite.

**Table 3:** Sensitivity and specificity for each category with each method.

Method/Category		1	2	3	4	5	6	7	8	9
VM-SVM	Sensitivity	0.9714	0.8857	1	0.8571	0.8286	1	0.6857	0.8286	0.7143
	Specificity	0.9857	0.9893	0.9964	0.9821	0.9786	1	0.9536	0.9857	0.9750
Platt’s method	Sensitivity	0.9714	0.8857	1	0.8571	0.8857	1	0.7143	0.8571	0.7714
	Specificity	0.9857	0.9893	0.9964	0.9893	0.9821	1	0.9607	0.9857	0.9786
VM-SR	Sensitivity	0.8000	0.8286	0.9429	0.7429	0.5714	1	0.5143	0.8000	0.8000
	Specificity	0.9714	0.9714	1	0.9536	0.9643	0.9964	0.9571	0.9750	0.9607
SR	Sensitivity	0.7429	0.7429	0.9714	0.7143	0.6000	1	0.5429	0.8000	0.7429
	Specificity	0.9714	0.9714	1	0.9536	0.9643	0.9964	0.9571	0.9750	0.9607
VM-NB	Sensitivity	0.8000	0	0.9714	0.6571	0.4286	0.9429	0.4286	0.4857	0.7143
	Specificity	0.8464	0.9786	0.9929	0.9500	0.9321	1	0.9429	0.9714	0.9393
NB	Sensitivity	0	0.3143	0.9714	0.2857	0.286	0.6857	0.571	0.3714	0.1429
	Specificity	1	0.9607	0.9929	0.8750	0.8786	0.9107	0.8929	0.8679	0.9500
